# Three‐dimensional reconstruction of testis cords/seminiferous tubules

**DOI:** 10.1002/rmb2.12413

**Published:** 2021-09-14

**Authors:** Hiroki Nakata, Shoichi Iseki, Atsushi Mizokami

**Affiliations:** ^1^ Department of Histology and Cell Biology Graduate School of Medical Sciences Kanazawa University Kanazawa Japan; ^2^ Department of Clinical Engineering Faculty of Health Sciences Komatsu University Komatsu Japan; ^3^ Department of Integrative Cancer Therapy and Urology Kanazawa University Graduate School of Medical Science Kanazawa Japan

**Keywords:** 3D, reconstruction, seminiferous tubule, serial section, testis cord

## Abstract

**Background:**

Due to the development of novel equipment for the acquisition of two‐dimensional serial images and software capable of displaying three‐dimensional (3D) images from serial images, the accurate 3D reconstruction of organs and tissues has become possible.

**Methods:**

Based on published studies, this review summarizes techniques for the 3D reconstruction of the testis cords/seminiferous tubules, with special reference to our method using serial paraffin sections and 3D visualization software.

**Main findings:**

The testes of mice, rats, and hamsters of various ages were 3D reconstructed and species and age differences in the structures of the testis cords/seminiferous tubules were analyzed. Our method is advantageous because conventional paraffin‐embedded normal and pathological specimens may be utilized for the 3D analysis without the need for complicated and expensive equipment.

**Conclusion:**

By further decreasing the time and labor required for the procedure and adding information on molecular localization, the technique for 3D reconstruction will contribute to the elucidation of not only the structures, but also the functions of various organs, including the testis.

## Graphical abstract

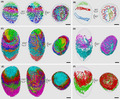

## 1 INTRODUCTION

Despite the development of advanced imaging technologies, traditional histology using tissue sections remains the gold standard for morphological tissue assessments in research and clinical practice. However, tissue sections obtained from three‐dimensional (3D) organs and tissues only provide two‐dimensional (2D) information. It is very important, but often challenging, to reconstruct missing 3D information in attempts to match the shape observed in a tissue section to the original 3D structure. Manual 3D reconstruction from serial sections is possible, but has seldom been used in morphological studies, possibly due to the extensive amount of labor required. In recent years, with the development of new technologies for the acquisition of 2D serial images and software capable of displaying 3D images from serial images, the accurate 3D reconstruction of organs and tissues has become possible.

Confocal microscopy,^1^, ^2^ one of the new imaging technologies, uses a laser beam and confocal pinholes to block out‐of‐focus light for the acquisition of 2D serial images with high resolution and contrast. The capturing of multiple 2D images at different depths in a tissue containing fluorescent molecules enables the reconstruction of 3D structures without the need for real tissue sections. However, the depth of tissue observable with confocal microscopy is restricted to 250–500 µm due to the attenuation of excitation light and fluorescence. Multiphoton excitation fluorescence microscopy^3^ and light sheet fluorescence microscopy^4^ increase the observable depth to a certain degree, but require complicated and expensive equipment. Micro‐computed tomography (micro‐CT)^5^, ^6^ is another imaging technology that creates virtual cross sections of organs and tissues with a micrometer‐level spatial resolution. It is composed of a rotating X‐ray tube and a row of detectors placed in the gantry to measure X‐ray attenuation. Multiple X‐ray measurements taken from different angles are then processed on a computer using reconstruction algorithms to produce tomographic images. Although the images obtained by micro‐CT are currently inferior to real histological sections because they lack color and have lower resolution and contrast, micro‐CT may become a useful tool for analyzing 2D and 3D structures in unfixed or fixed normal or pathological organs and tissues without their destruction.

In addition to the development of imaging technology, the advent of software that visualizes a 3D image from serial 2D images has been important for accurate 3D reconstruction.^7^ The key features of 3D visualization software are as follows: (1) image registration, which refers to the process of image alignment; (2) segmentation, which is the process of dividing multiple regions of interest in a 2D image; and (3) 3D rendering, which is a 3D computer graphic process that constructs a 3D model and visualizes it in a 2D image. The segmentation step is performed using automatic, semiautomatic, and manual tools. After 3D reconstruction, segmented regions may be used for a number of tasks, such as volumetric, density, and shape analyses. Due to the development of imaging technology and software, whole organs, such as the brain,^8^, ^9^, ^10^ kidneys,^11^, ^12^, ^13^ lungs,^14^ uterus,^15^ epididymis,^16^ ovaries,^17^, ^18^, ^19^, ^20^ and testes,^20^ have been analyzed in 3D at the micrometer level. This review provides a historical overview of techniques for the 3D reconstruction of the testis cords/seminiferous tubules, with special reference to our method using serial paraffin sections and high‐performance 3D visualization software.

## 2 MORPHOLOGY AND FUNCTIONS OF THE TESTIS

Mammalian testes consist of two compartments: the interstitial compartment and seminiferous tubule compartment.^21^, ^22^, ^23^ The former contains blood, lymphatic vessels, and nerves. The most frequent cell type in this compartment is the Leydig cell, which secretes testosterone.^24^, ^25^ Macrophages are also observed in the interstitial compartment and may play roles in the differentiation of spermatogonial stem cells.^26^, ^27^ The seminiferous tubule compartment is the site for spermatogenesis. There are 28 seminiferous tubules per testis for rats and 12 for mice on average,^28^, ^29^, ^30^ and they eventually connect to the rete testis while branching. Seminiferous tubules develop from fetal testis cords and consist of a seminiferous epithelium, which is divided into 14 stages in rats and 12 stages in mice according to germ cell associations.^30^, ^31^, ^32^, ^33^, ^34^, ^35^ Adjacent stages are aligned along a seminiferous tubule, and the time required for a particular stage to reappear in the same area is called the cycle, while the space occupied by a series of adjacent stages, including all possible types, is called the wave.^31^, ^36^, ^37^

A growing body of evidence indicates that region‐specific morphological and functional differences exist within the testis. For example, we recently revealed that the sites at which spermatids initially occur in the postnatal mouse testis are preferentially distributed in the upper‐medial areas of the testis close to the rete testis,^37^ and that the extent of markedly impaired spermatogenesis is significantly greater in tubule areas near the branching points in the mouse model of non‐obstructive azoospermia induced by the administration of busulfan.^38^ These regional differences may represent region‐specific gene expression that is regulated by particular molecules produced inside or outside the testis. Therefore, obtaining precise 3D information on the structure of the testis as well as cellular and molecular distributions provides a basis for investigations in various fields, including histology, pathology, and developmental biology.

## 3 METHOD FOR THE 3D RECONSTRUCTION OF TESTIS CORDS/SEMINIFEROUS TUBULES

The 3D structure of the whole testis was initially reported in the early 1900s^39^, ^40^, ^41^, ^42^; however, the majority of studies were on fetal testis cords and limited morphological information was available on the adult seminiferous tubules at that time. Curtis was the first to apply the technique of the 3D reconstruction of serial sections to adult mouse seminiferous tubules in 1918.^43^ The testes used were fixed in Flemming's solution, embedded in paraffin, cut into serial 10‐μm‐thick sections, and stained with iron hematoxylin. To analyze a whole tubule, a straight tubule opening into the rete testis was selected in a particular section and continuously followed through a series of sections. With an aid of the optical equipment available at the time, two complete adult mouse seminiferous tubules were manually reconstructed and one testis was estimated to contain 15 highly convoluted seminiferous tubules with three branching points. One and two seminiferous tubules in rabbit and dog testes, respectively, were also reconstructed.

A study by Clermont and Huckins in 1961 on the 3D structure of the rat testis cords/seminiferous tubules was the most impressive among the reconstruction studies using testis serial sections at that time.^29^ The testes used were fixed in Zenker's fluid, embedded in paraffin, cut into serial 5‐μm‐thick sections at intervals of 100 μm, and stained with periodic acid‐Schiff‐hematoxylin (PAS‐H). Plane figures on graph papers and 3D models constructed using stacked acrylic plastic sheets were both employed to visualize the structures of the testis cords/seminiferous tubules and elucidate their morphometric parameters. They reconstructed the rat testis cords on embryonic day (E) 17, E19, and postnatal day (P) 0 and analyzed their numbers and distributions. They also reconstructed 2 and 20 seminiferous tubules in P12 and adult rats, respectively. Two types of testis cords/seminiferous tubules were described: “outer cords/tubules” are in contact with the tunica albuginea, whereas “inner cords/tubules” are not. The next epoch‐making study on the 3D structure of mammalian testis cords/seminiferous tubules was not published until 2009, when two groups showed detailed 3D images of the mouse testis cords.^44^, ^45^

Combes et al. reconstructed the mouse testis cords on E12.25‐E15.5.^44^ Samples of the gonads were fixed in 4% paraformaldehyde (PFA) and, after whole‐mount fluorescent immunostaining for mesenchymal cells and basement membranes, observed on a Zeiss LSM 510 META inverted confocal microscope. Serial 5‐µm‐thick optical sections were taken throughout the depth of each sample. Two or more fields of view were required to provide the complete coverage of each sample. Optical sections were exported as an image series, manually aligned, and joined. Custom‐built macros generated in Adobe Photoshop (Adobe Inc.) were used to repeat the alignment and joining processes in the image series. The image stack was then imported into IMOD (http://bio3d.colorado.edu/imod/), a set of image processing, modeling, and display programs,^46^ with which the testis cords were segmented throughout the image stack using fluorescence and rendered to create a 3D representation.

Nel‐Themaat et al. investigated the structure of the testis cords in Sox9‐enhanced green fluorescent protein knock‐in mice by confocal microscopic imaging without reconstruction and by the 3D reconstruction of serial histological sections.^45^ Regarding cord reconstruction, gonads at E13.5 and E14.5 were fixed in 4% PFA, embedded in paraffin, serially sectioned at a thickness of 3 µm, and stained with hematoxylin and eosin. High resolution images of the sections were acquired every 9–15 µm and the testis cords were manually segmented in each image by marking their outlines in different colors using Adobe Illustrator software (Adobe Inc.). To generate 3D reconstruction, images were aligned using Adobe Photoshop CS2 (Adobe Inc.). A custom‐written MATLAB program (The Mathworks, Inc.) based on a linear algorithm was used to interpolate between the extracted sections. However, the 3D reconstruction of whole seminiferous tubules in the adult mammalian testis using a confocal microscope has not yet been performed. In the case of mice, the testis is an ellipsoid of 8 × 5 × 5 mm in size; therefore, it is difficult to observe the whole testis, even if made transparent and fluorescence‐stained, with confocal microscopy without making real histological sections.

Silva et al. recently investigated seminiferous tubules using micro‐CT.^47^ Mouse testes were fixed in 4% PFA, dehydrated, and stained with an alcohol‐based iodine agent to enhance soft tissue X‐ray contrast and prevent organ shrinkage during imaging. Samples were scanned on a Zeiss Xradia Versa 500 system (Oberkochen, Germany) with the X‐ray source operating with an anode voltage at 50 kV and power at 3 W. Images were reconstructed using volume reconstruction software integrated in the Xradia machine and 3D renderings were made using the commercial software VGstudio (Volume Graphics GmbH). Morphometry was performed using the Phong or Scatter HQ algorithm. Using this method on a 4‐week‐old mouse testis, they obtained micro‐CT images that were equivalent to conventional histological sections and a 3D rendering of the surface of seminiferous tubules in contact with the tunica albuginea. However, the reconstruction of whole seminiferous tubules was not conducted.

We recently reconstructed whole testis cords/seminiferous tubules using serial sections and Amira 6.3.0 (Visage Imaging GmbH), high‐performance 3D reconstruction software.^30^, ^37^, ^38^, ^48^, ^49^ In mice, the testis and epididymis were dissected out *en bloc* and fixed in Bouin's solution overnight at room temperature. Serial 5‐µm‐thick sections at intervals of 50 µm were obtained by cutting the specimen longitudinally in parallel to the plane involving both the testis and epididymis using a microtome and stained in the basement membrane with PAS‐H to mark the outlines of seminiferous tubules. Sections were digitized using a whole‐slide scanner (Nanozoomer 2.0‐HT; Hamamatsu Photonics) with a 20‐fold objective lens, and the resulting digital images of sections were visualized using viewer software (NDP.view2; Hamamatsu Photonics). The extraction of the PAS‐H‐stained basement membrane in digital images was performed using ImageJ software (NIH; http://imagej.nih.gov/ij/) and Adobe Photoshop 2020 software (Adobe Inc.). After extraction, images were converted into gray scale in the JPEG format with Adobe Photoshop 2020 software. Using Amira 6.3.0 software, serial images were automatically aligned with rigid algorithms followed by manual adjustments, and the inside of the outlines of a selected tubule was filled with a particular color using threshold processing and traced from section to section. This procedure, called semiautomatic segmentation, was repeatedly applied to all seminiferous tubules with different colors, and they were then subjected to 3D rendering. To draw the core lines of seminiferous tubules, individual traced tubules in cross sections were shrunk concentrically and reconstructed into a thin tubule, in which the core lines were drawn using the same software. The software sometimes failed to distinguish closely apposed different tubules from the branched portions of a single tubule and drew wrong lines, partly because alignment was slightly inaccurate due to the distortion of sections. This error was corrected manually by tracking the reconstructed core lines in all serial sections. While the method described above was the standard, we also made some modifications to it depending on the specimens and fixatives. For example, small and soft specimens, such as embryonic gonads, were fixed in modified Davidson's fluid or 10% formalin neutral buffer solution, a milder fixative than Bouin's solution, and the basement membranes were immunostained for marker proteins, such as laminin, to enable more accurate semiautomatic segmentation. On the other hand, large specimens, such as the adult hamster testis, were sometimes insufficiently fixed, even with Bouin's solution, making PAS‐H staining of the basement membrane not sufficiently clear to allow for semiautomatic segmentation. In such cases, we adopted manual segmentation by marking the outlines of seminiferous tubules from section to section. Using these methods, we reconstructed the testis cords/seminiferous tubules of mice, rats, and hamsters of various ages using serial paraffin sections.^30^, ^37^, ^38^, ^48^, ^49^

## 4 3D IMAGING OF TESTIS CORDS/SEMINIFEROUS TUBULES USING SERIAL SECTIONS

**FIGURE 1:**
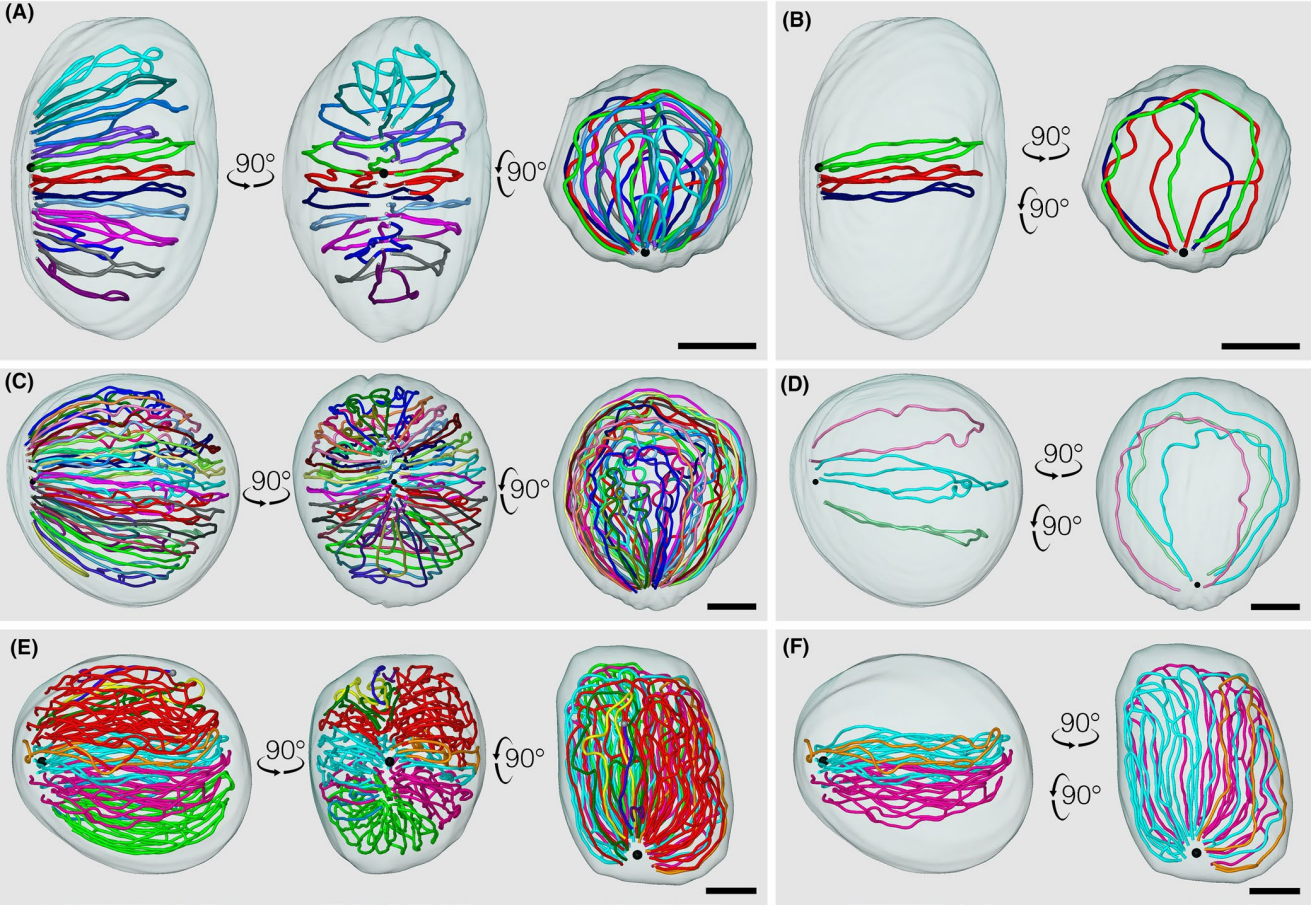
Core lines of individual reconstructed testis cords/seminiferous tubules from an E15.5 mouse (A, B), E19.5 rat (C, D), and P0 hamster (E, F) are marked in different colors and superimposed on the reconstructed testis by orthographic projections. All (A, C, E) or representative (B, D, F) testis cords/seminiferous tubules are viewed from 3 (A, C, E) or 2 (B, D, F) directions. The position of the rete testis is shown in a black sphere. All scales, 200 µm [Colour figure can be viewed at wileyonlinelibrary.com]

Figure 1 shows the 3D structure of the testis cords soon after the onset of coiling in representative testes of mice, rats, and hamsters.^30^, ^49^ In E15.5 mice, the average numbers of testis cords and branching points per testis were 12.3 and 21.0, respectively (n = 3). In contrast, in E19.5 rats, the average numbers of testis cords and branching points per testis were 29.7 and 3.3 (n = 3), which were significantly larger and smaller, respectively, than those in mice. In P0 hamsters, the average numbers of seminiferous tubules and branching points per testis were 9.0 and 93.0 (n = 3), which were significantly smaller and larger, respectively, than those in mice. Therefore, the numbers of testis cords and branching points per testis were inversely related among the 3 species. This is presumably due to differences among the 3 species in the frequencies of the fusion of neighboring testis cords during the earlier process of testis formation, which increases the number of branching points and decreases the number of testis cords/seminiferous tubules. In relation to this, Clermont & Huckins^29^ classified the types of testis cords/seminiferous tubules in rats into “inner” and “outer,” with the former having no contact with the tunica albuginea. In contrast, we found that most of the testis cords/seminiferous tubules in mice and hamsters were in contact with the tunica albuginea, similar to the outer cords in rats. Therefore, we considered it inappropriate to separate the outer and inner types in mice and hamsters. This phenomenon is presumably interpreted by the lower frequency of the fusion of testis cords in rats. On the other hand, we found that seminiferous tubules in hamsters may be classified into “shorter” and “dominant” types. Dominant‐type cords, 2–4 per testis and accounting for more than 80% of the total cord length, have numerous branching points, suggesting that they are formed by the extensive fusion of preexisting testis cords during the earlier process of testis formation in hamsters.

**FIGURE 2:**
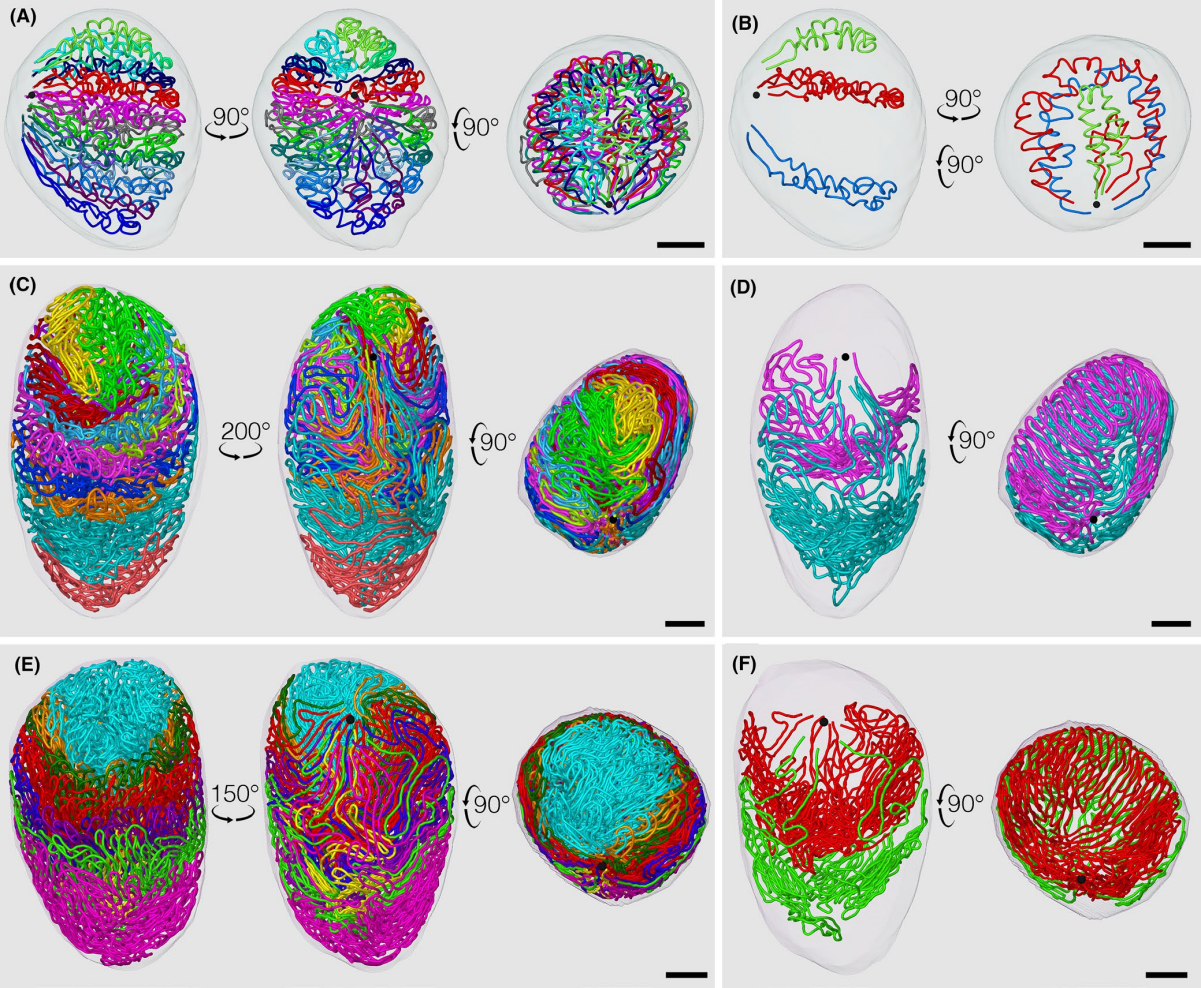
Core lines of individual reconstructed testis cords/seminiferous tubules from E18.5 (A, B), P21 (C, D), and adult (E, F) mice are marked in different colors and superimposed on the reconstructed testis by orthographic projections. All (A, C, E) or representative (B, D, F) testis cords/seminiferous tubules are viewed from 3 (A, C, E) or 2 (B, D, F) directions. The position of the rete testis is shown in a black sphere. Scales; 200 µm (A, B); 500 µm (C, D); 1 mm (E, F) [Colour figure can be viewed at wileyonlinelibrary.com]

Figure 2 shows the 3D structure of the testis cords/seminiferous tubules in E18.5, P21, and adult mice.^30^, ^37^ On E18.5, testis cords were extensively coiled and their total length was 59.3 mm, which was 3.2‐fold larger than that on E15.5 (18.5 mm). The cords on the cranial side were strongly coiled, whereas those on the caudal side were only weakly coiled. Increases in the testis volume and length of the testis cords are important factors for explaining this phenomenon. During the final stages of testis development, the caudal side of the testis grew faster than that of the cranial side, resulting in the upward movement of the position of the rete testis. Since the elongation speed of the testis cords does not significantly differ between the cranial and caudal sides, the relative lack of space necessary for the elongation of the testis cords may lead to the earlier onset and progression of the coiling of cords on the cranial side of the testis. One of the reasons for the greater expansion of the testis volume on the caudal side may be the regional difference in the plasticity of the tunica albuginea.

**FIGURE 3:**
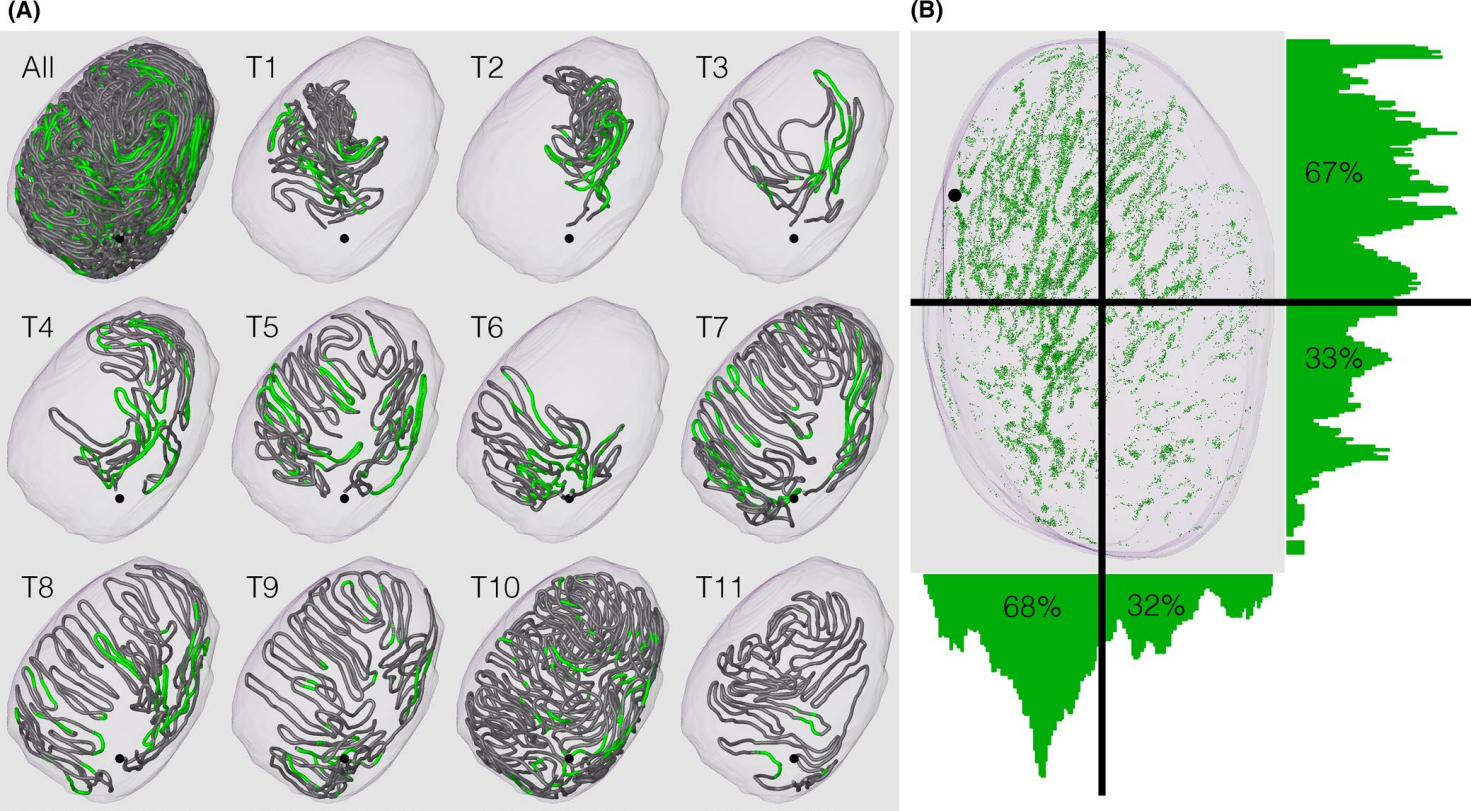
Distributions of acrosomes visualized with the lectin peanut agglutinin in the testis of P21 mice. (A) The core lines of all reconstructed seminiferous tubules named T1–T11 in the order of their connections with the rete testis are shown in a horizontal view with the portions of tubules containing acrosomes marked in green. (B) Reconstructed acrosomes are shown as green dots and superimposed on the testis in a frontal view. The relative volumes of acrosomes in 20‐µm‐thick slices of a testis against the volumes of respective slices are plotted in bar graphs. The numerals shown in the graphs represent the volumes of acrosomes in the areas of the testis against the total volume of acrosomes in the testis (%). The position of the rete testis is shown in a black sphere [Colour figure can be viewed at wileyonlinelibrary.com]

In P21 mice, the zigzag convolutions of seminiferous tubules became more constant in direction. The cranial turns of convolutions were in contact with the tunica albuginea, whereas the caudal turns occupied the central areas of the testis. Overall, all seminiferous tubules formed funnel‐shaped networks that tapered toward the caudal direction, and caudally located networks surrounded the preceding cranially located networks from the bottom and outside with the appearance of stacked paper cups. The acrosome, a special structure of the spermatid that appears for the first time in the P21 mouse testis, was visualized with lectin histochemistry and superimposed onto the testis and seminiferous tubules at P21 (Figure 3). Acrosomes were distributed more preferentially in cranial than in caudal seminiferous tubules within the testis and in portions proximal rather than distal to the rete testis within seminiferous tubules. Assuming that the speed of the progression of spermatogenesis does not significantly differ along the length of the seminiferous tubules, these portions represent the sites at which the first round of spermatogenesis begins in the early postnatal period. The biological meaning of the preferential distribution of the onset of spermatogenesis currently remains unknown, but may be related to the efficiency of spermatozoa transport to the rete testis.

In adult mice, the total length of seminiferous tubules per testis was 86‐, 27‐, and 2.2‐fold larger than those in E15.5, E18.5, and P21 mice, respectively (Figure 2). Each tubule ran along a circular path within the testis while making convolutions with cranial and caudal hairpin turns. The cranial turns of all tubules were in contact with the tunica albuginea, whereas the caudal turns were apart from it, resulting in funnel‐shaped networks of these tubules with tapered caudal portions. This pattern in the paths of seminiferous tubules was already established by P21, but became clearer in adults. Although testis cords/seminiferous tubules showed marked variations between individual mice, their basic structures were similar and retained from E15.5 to adults. Among the 28 mouse testes analyzed in our previous studies at ages ranging from E15.5 to adult, the average numbers of testis cords/seminiferous tubules, terminating points (connections with the rete testis), and branching points per testis were 12.1 ± 1.5 (range, 9–16), 39.6 ± 4.6 (range, 28–49), and 16.5 ± 4.3 (range, 9–24), respectively. Among the 340 testis cords/seminiferous tubules reconstructed, 124 (36%) were simple cords/tubules without any branching points, 107 (31%) had one branch, 109 (32%) had two or more branches, and only nine (3%) had a blind end.

## 5 PERSPECTIVES

Accurate 3D information at the cellular and tissue levels is becoming increasingly important in research and clinical practice. Despite the development of advanced imaging technologies, such as confocal microscopy and micro‐CT, an abundant amount of information is still obtained from classical histological sections. In this review, we demonstrated the 3D reconstruction of testis cords/seminiferous tubules using serial histological sections and 3D visualization software. This method is advantageous because conventional paraffin‐embedded normal and pathological specimens, the accumulation of which is extensive in ordinary histology and pathology laboratories, may be utilized for 3D analyses without the need for complicated and expensive equipment.

The limitations of this method are that it requires time and labor, preventing its application to the analysis of larger specimens, such as the human testis. The segmentation and tracing of seminiferous tubules in serial sections, even if performed semiautomatically using PAS‐ or immunolabeled basement membranes, remain the most time and labor‐consuming steps. However, the use of emerging technology on artificial intelligence (AI) based on the deep learning of pattern recognition will overcome this issue by automating both the segmentation and tracing steps. We have started analyzing the 3D structure of the human testis using this AI technology.

Furthermore, as already discussed, region‐specific morphological and functional differences within the testis may represent region‐specific gene expression that is regulated by particular molecules produced inside or outside the testis. The histochemical localization of various bioactive molecules and their receptors may be superimposed on the 3D‐reconstructed testis structure, similar to the lectin that recognized acrosomal molecules in our previous study.^37^ With the addition of this molecular information, 3D reconstruction will contribute to the elucidation of not only the structures, but also the functions of various organs, including the testis.

## CONFLICTS OF INTEREST

The authors declare no conflicts of interest. The present animal study was approved by Kanazawa University (approval number: AP‐153636 and AP‐173897), the Tokyo Medical University Committee (R2‐0045), and the Animal Research Committee of St. Marianna University School of Medicine (1712012).
